# Rheological Behaviors and Damage Mechanism of Asphalt Binder under the Erosion of Dynamic Pore Water Pressure Environment

**DOI:** 10.3390/polym14214731

**Published:** 2022-11-04

**Authors:** Wentao Wang, Kang Zhao, Tingting Xie, Huifang Liu, Guanyi Zhao, Linbing Wang

**Affiliations:** 1 National Center for Materials Service Safety, University of Science and Technology Beijing, Beijing 100083, China; 2 The Sensing and Perception Lab, School of Environmental, Civil, Agricultural and Mechanical Engineering, University of Georgia, Athens, GA 30602, USA

**Keywords:** asphalt binder, moisture damage, dynamic pore water pressure, rheological behaviors, damage mechanism

## Abstract

Asphalt binder plays an important role in the overall resistance of asphalt mixture to the moisture damage induced by a dynamic pore water pressure environment. This study evaluates the moisture sensitivity of asphalt binder from the perspective of rheological behaviors using the dynamic shear rheometer (DSR) and the bending beam rheometer (BBR) methods at high, medium, and low temperatures. The damage mechanism is further discussed quantitatively based on the Fourier transform infrared spectroscopy (FTIR) method. The results indicate that a longer conditioning duration is beneficial for asphalt binder to recover its adhesion at 60 °C in multiple stress creep recover (MSCR) tests, but the increasing pore water pressure magnitude of 60 psi held an opposite effect in this study. The asphalt binder’s fatigue life at 20 °C in linear amplitude sweep (LAS) tests decreased obviously with conditioning duration and environmental severity, but the reducing rate gradually slowed down, while the groups of 50 psi—4000 cycles and 60 psi—4000 cycles held a comparable erosion effect. Both the stiffness and relaxation moduli at −12 °C in the BBR tests exhibited an obvious decreasing trend with conditioning duration and environmental severity. The erosion effect on the asphalt binder was gradually enhanced, but it also exhibited a slightly more viscous performance. Water conditioning induced several obvious characteristic peaks in the FTIR absorbance spectra of the asphalt binder. The functional group indexes presented a trend of non-monotonic change with conditioning duration and environmental severity, which made the asphalt binder show complicated rheological behaviors, such as non-monotonic variations in performance and the abnormal improving effect induced by dynamic pore water pressure conditioning.

## 1. Introduction

Asphalt binder plays a vital role in bonding aggregate particles with different sizes, mineral powder, and additives tightly together to form an asphalt mixture, such as hot mix asphalt (HMA). For asphalt pavement located in rainy regions, its service performance is severely challenged by the environment of dynamic pore water pressure formed by the interaction between moving vehicle tires, surface runoff, and asphalt pavement [1], which causes moisture damage and affects its service life. Specifically, the comprehensive service performance of the eroded asphalt pavement is undoubtably affected at different temperatures [2]. Different from traditional water environments, such as freeze–thaw and static water immersion [3], the erosion processes in a dynamic pore water pressure environment exhibit quite a unique damage mechanism on asphalt binder. In this case, it is meaningful to explore the performance of asphalt binder, such as rheological behaviors, in the erosion of a dynamic pore water pressure environment and make efforts to investigate this relevant damage mechanism.

Multiple techniques have been adopted to explore the moisture damage of asphalt binder. The dynamic shear rheometer (DSR) technique is a quite useful method to inspect the properties of asphalt binder, due not only to its rich mechanical loading modes but also to the advantage of less consumption of asphalt binder samples. Jing et al. [4] investigated the effect of aging on the viscoelastic characteristics of asphalt binder which mainly include rheological, fatigue, and relaxation properties. Zhang and Gao [5] predicted fatigue crack growth in viscoelastic asphalt binder and presented a new model named DSR-C for prediction. Das and Singh [6] studied the influence of nano-size hydrated lime filler on the rutting performance of asphalt mastic. Ziade et al. [7] combined DSR and numerical techniques to investigate rheological behavior in asphalt binder composites. It is convenient to adopt the DSR method to evaluate the rheological behaviors of asphalt binder before and after being conditioned in a dynamic water environment.

The bending beam rheometer (BBR) test can measure the flexural creep performance of asphalt binder at a relative low temperature below 0 °C. Basically, creep stiffness *S* and the flexural creep rate *m-value* are often used to assess the performance of asphalt binder [8,9,10]. However, more viscoelastic properties can be further explored, such as master curve of modulus, creep compliance, and relaxation modulus, based on the measured raw data in a BBR test [11]. In particular, the indicator of the ratio of creep rate *m-value* to creep stiffness *S* was found to represent the change rate of creep compliance [12], which could be applied to evaluate the erosion degree of the moisture damage of asphalt binder.

The Fourier transform infrared spectroscopy (FTIR) technique can detect the change in the functional groups and chemical bonds of asphalt binder before and after a certain environment conditioning. Ahmad et al. [13] used the FTIR method to assess the chemical and mechanical changes in asphalt binder due to moisture conditioning and found an increase in the hydroxyl group. Mannan et al. [14] combined the FTIR and DSR methods to investigate the healing properties of asphalt binder after being eroded by a dynamic water environment.

Based on the analysis discussed above, it could be found that systematic research on moisture damage related to a dynamic pore water pressure environment on asphalt binder’s rheological behaviors was not sufficient, especially at the various ranges of low, medium, and high temperatures. For the FTIR technique, it is essential to quantitatively analyze characteristic peaks with their nearby regions together, but not only to compare the relative position of the infrared spectra qualitatively, which might be helpful to explore the mechanism of moisture damage.

The main objective of this study is to evaluate the moisture sensitivity of asphalt binder from the perspective of rheological behaviors and its damage mechanism. The parameters of conditioning duration and pore water pressure magnitude were taken into account to provide different types of dynamic water conditioning environments. The DSR method was first applied to evaluate the high-temperature property and the medium-temperature fatigue performance for the conditioned asphalt binder. Then, the BBR method was adopted to explore the retained low-temperature creep properties and the change in the viscous performance of the asphalt binder. Finally, the FTIR method was selected to quantitatively investigate the fluctuations of the characteristic peaks in the infrared spectra.

## 2. Materials and Sample Preparation

In this study, a typical base asphalt binder with the performance grade (PG) of 64−16 was selected to be conditioned in a dynamic pore water pressure environment, and then samples were prepared for the DSR, BBR, and FTIR experiments. The basic physical properties of the asphalt binder were inspected, and the relevant results are summarized in Table 1, which meet the requirements of the specification [15].

To thoroughly erode the samples, asphalt binder was first poured into a shallow disc container with the thickness controlled at about 4 mm and then placed in a dynamic pore water pressure environment for full conditioning. The conditioned asphalt binder was then heated in a 100 °C oven environment to evaporate possible retained water, followed by sample fabrications in a flow state for further residual performance inspection. For the DSR tests, the asphalt binder was poured into the molds with diameters of 8 mm and 25 mm, and two parallel samples were applied for each DSR test. For the BBR tests, thin beam samples were made, and their dimensions were controlled at 127 ± 2 mm in length, 12.7 ± 0.05 mm in width, and 6.35 ± 0.05 mm in thickness, while three parallel samples were applied. Further, asphalt-binder-coated film slides were made for the FTIR inspection.

## 3. Test and Analysis Methods

### 3.1. Dynamic Pore Water Pressure Conditioning Method

**Figure 1:**
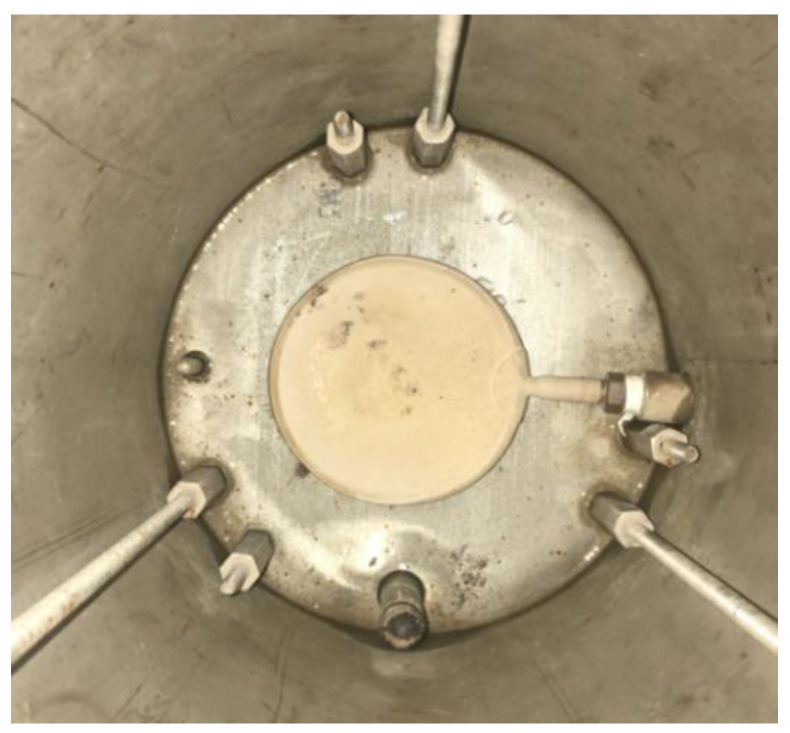
A bladder inside the sealed container of MIST.

The device named Moisture Induced Sensitivity Tester (MIST) was selected to provide the water conditioning environment of dynamic pore water pressure in this study [16]. A typical bladder located in the sealed cylinder container of the MIST device is driven to be repeatedly inflated and restituted, as shown in Figure 1, which can provide the cyclic water conditioning environment of dynamic pore water pressure. Three variable parameters can be adjusted in the MIST method, which include pore water pressure magnitude, water temperature, and conditioning duration. In this study, the water temperature was controlled at 60 °C to ensure a stable and typical high-temperature water environment for all sample conditioning tests. The variable parameter of conditioning duration was adjusted to 2000, 3000, and 4000 cycles for the water environment, with the pore water pressure magnitude controlled at 50 psi. In the MIST test, it takes about 4 s for each conditioning cycle of the dynamic pore water pressure environment. Further, an experimental group was set to the 60 psi pore water pressure magnitude with the conditioning duration controlled at 4000 cycles.

### 3.2. The DSR Method

**Figure 2:**
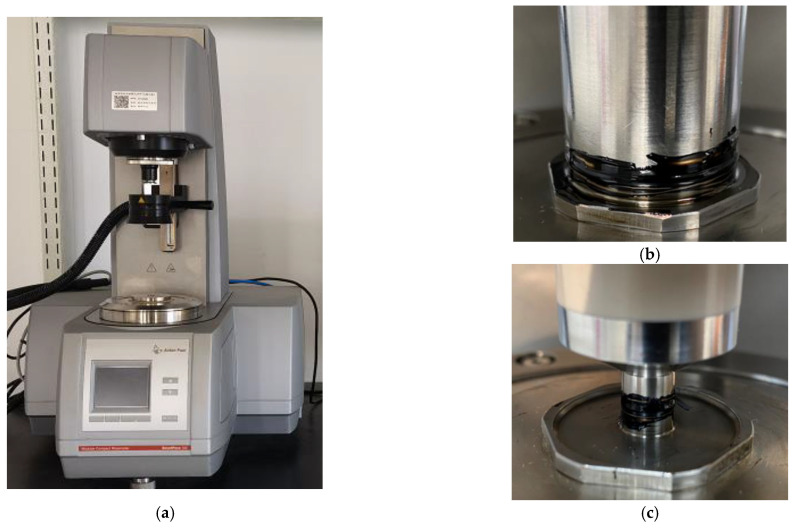
The DSR test: (**a**) The DSR device; (**b**) A sample with 25 mm dia.; (**c**) A sample with 8 mm dia.

The DSR method was applied to evaluate the rheological behaviors of asphalt binder before and after being conditioned in a dynamic pore water pressure environment. Two types of typical DSR tests were conducted in high and medium temperatures, which included the multiple stress creep recover (MSCR) test and the linear amplitude sweep (LAS) test. The DSR device used in this study and the experimental details are shown in Figure 2.

#### 3.2.1. MSCR Test

**Figure 3:**
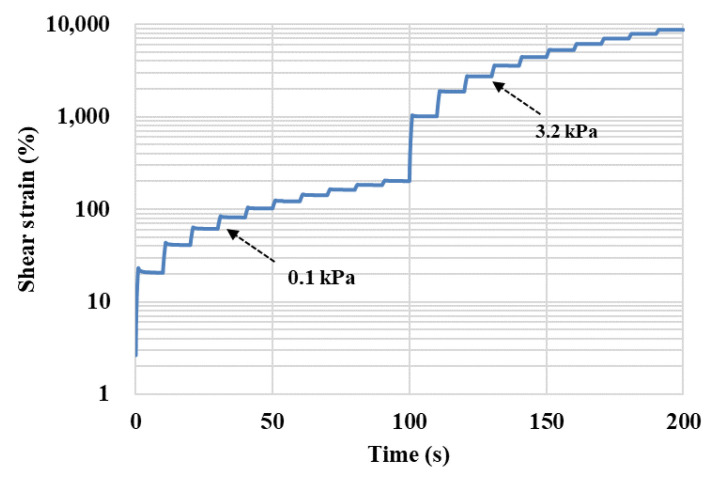
A typical shear strain curve in a MSCR test.

The MSCR tests were conducted to evaluate the change in the asphalt binder’s performance at the high temperature of 60 °C after being conditioned in a dynamic water environment. A MSCR test contains 20 cyclic cycles, in which the first 10 cycles apply a creep stress of 0.1 kPa and the remaining 10 cycles load at 3.2 kPa. Each cycle includes a loading period of 1 s followed by a recovering period of 9 s, while the entire MSCR test lasts for 200 s [17]. A typical shear strain curve in a MSCR test is shown in Figure 3. The indicator of unrecoverable creep compliance *J_nr_* for each creep recovery cycle can be calculated based on Equation (1), where *γ_nr_* and *γ*_0_ are the initial strain and residual strain for each creep-recovery cycle and *τ* is shear stress. The average value of *J_nr_* is obtained based on 10 cycles for each shear stress level.

(1) $$J_{nr}=\frac{\gamma_{nr}-\gamma_{0}}{\tau }$$

#### 3.2.2. LAS Test

**Figure 4:**
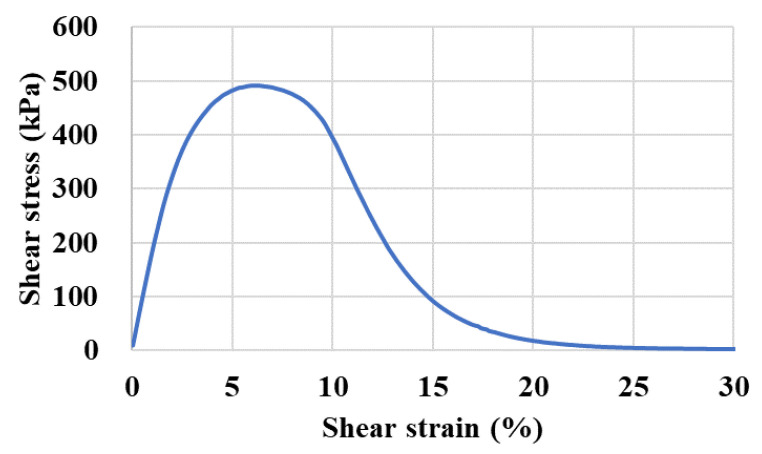
A typical shear strain-stress curve in a LAS test.

The LAS tests were adopted to investigate the variation in fatigue behavior at the medium temperature of 20 °C for the asphalt binder before and after being conditioned in a dynamic water environment. The applied shear strain with a sinusoidal wave is controlled in the LAS test, which increases linearly from 0.1% to 30% [18]. The load frequency was set to 10 Hz. A typical curve between shear strain and stress is shown in Figure 4. A simplified viscoelastic continuum damage (S−VECD) model was selected to obtain the damage characteristic curve to evaluate the evolution of the fatigue damage of the asphalt binder. Equation (2) can be applied to fit the fatigue damage characteristic curve between the damage variable *S* and pseudo-modulus *C*, in which *C*_0_ is often set at 1 and both *C*_1_ and *C*_2_ are curve-fit coefficients.

(2) $$C=C_{0}-C_{1}{\left(S\right)}^{C_{2}}$$

### 3.3. The BBR Method

**Figure 5:**
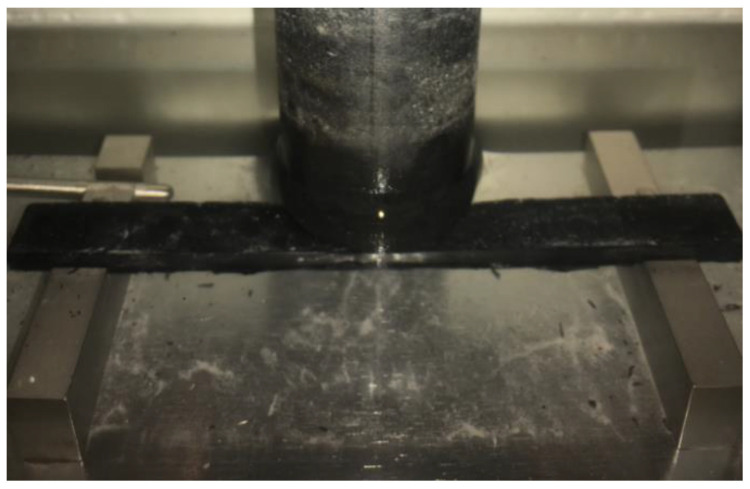
A sample of asphalt binder in the BBR test.

The BBR method was applied to inspect the low-temperature rheological behaviors of asphalt binder before and after being eroded by a dynamic pore water pressure environment. According to the ASTM D6648 standard [19], as shown in Figure 5, a thin beam sample of asphalt binder is placed on two supports with a constant load of 980 ± 50 mN located at its mid-span, while the flexural deformation can be further measured. In this study, the low temperature of the BBR tests was controlled at −12 °C according to the PG 64−16 of the asphalt binder.

In the BBR method, the basic indicators of the flexural creep stiffness *S* and the flexural creep rate *m-value* can be calculated based on the measured creep mechanical stress and strain data. The flexural creep stiffness *S*(*t*) is determined using Equation (3), where *δ(t)* is the deformation at the mid-span of the thin beam sample, *P* is the constant load, and *h*, *L*, and *b* are the thickness, length, and width of the thin beam sample. The flexural creep rate *m*(*t*) is the absolute value of the slope of the flexural creep stiffness *S*(*t*) versus time curve in the double logarithm coordinate system. In particular, creep compliance *D* shares a countdown relationship with flexural creep stiffness modulus *S*, which can be further applied to explore more rheological behaviors of asphalt binder via the BBR test.

(3) $$S\left(t\right)=\frac{PL^{3}}{4bh^{3}\delta \left(t\right)}$$

**Figure 6:**
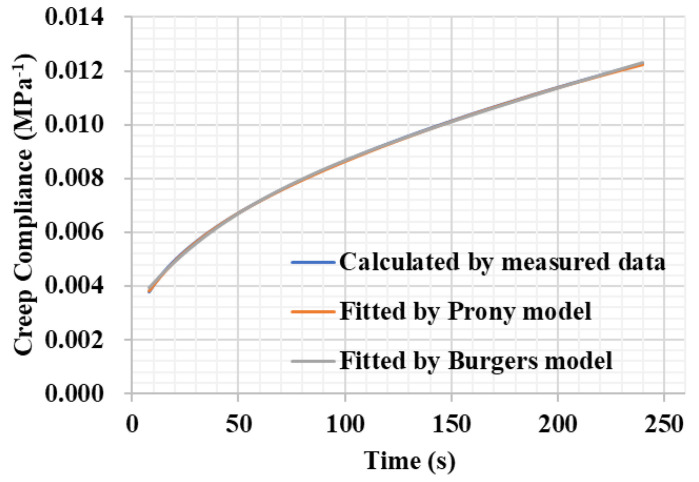
Creep compliance curves determined using different methods in a BBR test.

As shown in Equations (4) and (5), both the Burgers model and the second-order Prony series expression can represent the creep compliance *D*(*t*) of asphalt binder according to the BBR test. The Burgers model takes characteristics of the Maxwell and Kelvin models into account, while the second-order Prony series expression was constructed based on the generalized Kelvin model. As shown in Figure 6, both the Burgers and Prony models can well fit the creep compliance curve in the BBR test.

(4) $$D\left(t\right)=\frac{\varepsilon \left(t\right)}{\sigma_{o}}=\frac{1}{E_{1}}+\frac{t}{\eta_{1}}+\frac{1}{E_{2}}\left(1-e^{-t\cdot \frac{E_{2}}{\eta_{2}}}\right)$$

(5) $$D\left(t\right)=D_{0}+D_{1} [1-\exp\left(-\frac{t}{\rho_{1}}\right)]+D_{2} [1-\exp\left(-\frac{t}{\rho_{2}}\right)]$$

In the Burgers model, *E*_1_ and *E*_2_ are the instantaneous elastic modulus of the Maxwell model and the delayed elastic modulus of the Kelvin model, respectively, while both *η*_1_ and *η*_2_ are viscous coefficients. These viscoelastic parameters can further calculate the indexes of relaxation time *λ* and the delay time *τ*, which are determined using Equations (6) and (7). Relaxation time *λ* shows the ability of asphalt binder to release internal stress, and delay time *τ* often indicates creep and delayed elastic performance. A shorter relaxation time *λ* often means a better viscous performance, while a larger *τ* usually indicates a much more viscous deformation, which means a good anti-cracking ability at a low temperature.

(6) $$\lambda =\frac{\eta_{1}}{E_{1}}$$

(7) $$\tau =\frac{\eta_{2}}{E_{2}}$$

In the second-order Prony series expression, *D*_0_ is the compliance of the individual spring element in the Kelvin model, *D*_1_ and *D*_2_ are the compliances of the spring element in a Kelvin element, and *ρ*_1_ and *ρ*_2_ are ratios of the viscosity coefficient of a dashpot element to the modulus of the spring element in a Kelvin element. As creep compliance *D* has a reciprocal relationship with relaxation modulus *E* in the Laplace domain, as shown in Equation (8), the relaxation modulus *E* can be thus derived, and the detailed derivation processes can be found in reference [20].

(8) $$\int_{0}^{t}E\left(t-\tau \right)\frac{\partial D\left(t\right)}{\partial \tau }d\tau =1$$

In particular, an indicator *m*/*S* can be derived to reflect the growth rate of creep compliance *D*′(*t*), as shown in Equation (9), which combines characteristics of both the creep stiffness *S* and creep rate *m-value*. The detailed derivation processes of the *m*/*S* indicator can be found in the previous research [12]. Time *t* is often set at a representative 60th s, and a larger *m*(*t*)/*S*(*t*) often indicates a larger *D*′(*t*), which means a slower increase in creep stiffness.

(9) $$\frac{m\left(t\right)}{S\left(t\right)}\approx D^{\prime }\left(t\right)\times t$$

### 3.4. The FTIR Method

The FTIR method can effectively inspect the change in the functional groups and chemical bonds of asphalt binder after being eroded by external service environments such as a dynamic pore water pressure environment. The moisture damage mechanism can be illustrated to a certain extent based on the several appeared obvious characteristic peaks in several ranges of wavenumbers of the obtained infrared spectrum.

Based on the measured transmissivity curves, the absorbance curves with a wavenumber range from 4000 to 400 cm^−1^ for the different types of conditioned asphalt binder samples can be determined. After being calibrated based on the baseline and subsequently smoothed, the area values of these obvious characteristic peaks for the functional groups and chemical bonds of the asphalt binder in an absorbance curve were calculated. In particular, the area of a characteristic peak was determined according to the tangent line connected by its lowest points on the left and right sides. In order to eliminate the influence of possible experimental errors as much as possible, the relative area rather than the raw area was selected for further analysis. As the characteristic peak around the 2923 cm^−1^ wavenumber appeared in the infrared spectra for all types of asphalt binder samples, the area of this characteristic peak was selected as the base functional group area to determine the relative area values of the other functional groups and chemical bonds for all types of asphalt binder. Therefore, a characteristic functional group index *I_i_* for asphalt binder can be thus determined using Equation (10), in which *A_i_* is the relative area for a characteristic peak and *ΣA_i_* is the sum of these relative areas of all occurred characteristic peaks.

(10) Ii=Ai∑Ai

## 4. Results and Discussion

### 4.1. High-Temperature Rheological Behavior

**Figure 7:**
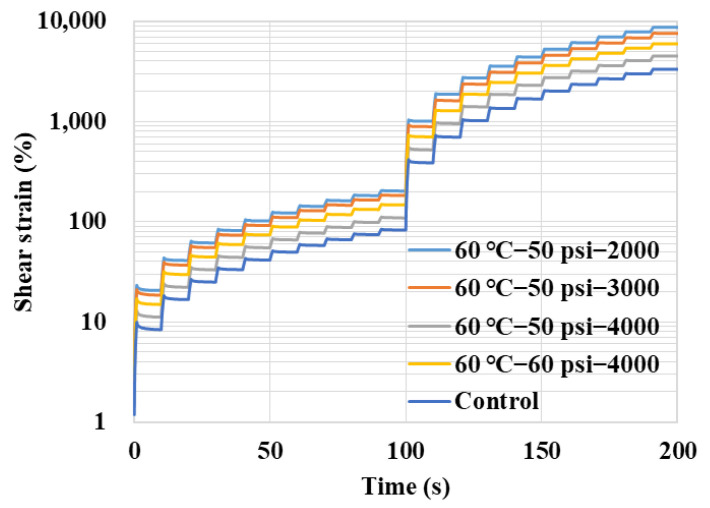
Shear strain curves in MSCR tests.

The shear strain curves for the asphalt binder conditioned in different dynamic water environments are summarized in Figure 7. It could be found that the curves for the conditioned asphalt binder are located in relatively higher positions compared with the control group, which indicates the decline in high-temperature performance after being eroded by dynamic water. In the dynamic water environment of 60 °C−50 psi, the shear strain curves gradually moved down with the increase in conditioning duration, which showed a decreasing accumulated permanent deformation. Compared between the groups of 50 psi and 60 psi, it could be found that the shear strain curve of the 50 psi group was located in a relatively lower position, which means an increasing deformation and a worse high-temperature creep recovery ability of the asphalt binder with the increase in environment severity.

**Figure 8:**
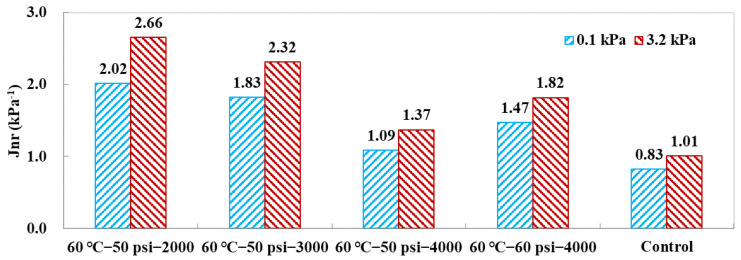
Variation of unrecoverable creep compliance *Jnr* in MSCR tests.

As a standard indicator, *J_nr_* can well evaluate the ability to resist the high-temperature rutting problem of asphalt binder. The variations of unrecoverable creep compliance *J_nr_* are summarized in Figure 8, which include *J*_*nr*0.1_ and *J*_*nr*3.2_ for the two shear stress levels. It could be found that both *J*_*nr*0.1_ and *J*_*nr*3.2_ decreased with the conditioning duration, which exhibited an improved effect on the high-temperature performance of the asphalt binder. A longer conditioning duration would be beneficial for asphalt binder to recover its adhesion. However, the increasing pore water pressure magnitude of 60 psi held an opposite effect. Therefore, a suitable combination of pore water pressure magnitude and conditioning duration would be helpful for asphalt binder to increase its cohesion to aggregate.

### 4.2. Medium-Temperature Fatigue Behavior

**Figure 9:**
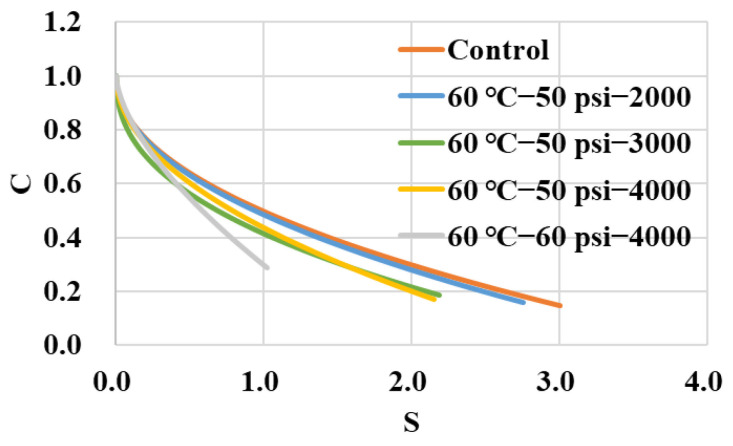
Damage characteristic curves of asphalt binder in LAS tests.

After being conditioned in dynamic pore water pressure, the retained fatigue behaviors of the asphalt binder were evaluated at the medium temperature of 20 °C using the LAS method. The damage characteristic curves of these samples of asphalt binder are presented in Figure 9. Compared with the control group, the indicator of pseudo-modulus *C* decreased with the conditioning duration and environmental severity at a certain same damage variable *S*, which indicated an increasing evolution of fatigue damage. To reach a certain pseudo-modulus *C*, the group of 60 °C−60 psi−4000 cycles held the relative smallest damage variable *S*, which showed the severe erosion of the dynamic water environment on the asphalt binder.

**Figure 10:**
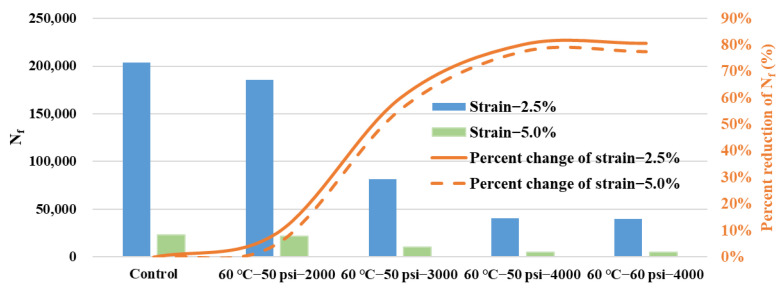
Fatigue life of asphalt binder in LAS tests.

The *C-S* curve can illustrate the basic characteristics of the fatigue damage evolution of asphalt binder during a LAS load, while fatigue life *N_f_* can be thus determined to evaluate the fatigue performance in different levels of shear strains. Fatigue life *N_f_* of these water-conditioned samples are predicted and summarized in Figure 10. At the shear strains of both 2.5% and 5.0%, the indicators of fatigue life *N_f_* for the asphalt binder samples conditioned in different dynamic water environments exhibited an obvious decreasing trend with conditioning duration and environmental severity. The percent reduction of fatigue life *N_f_* was also determined. It was found that the rate of reduction slowed down, especially for the groups of 50 psi−4000 cycles and 60 psi−4000 cycles, which means a comparable erosion effect by the dynamic water environments.

### 4.3. Low-Temperature Flexural Creep Behavior

**Figure 11:**
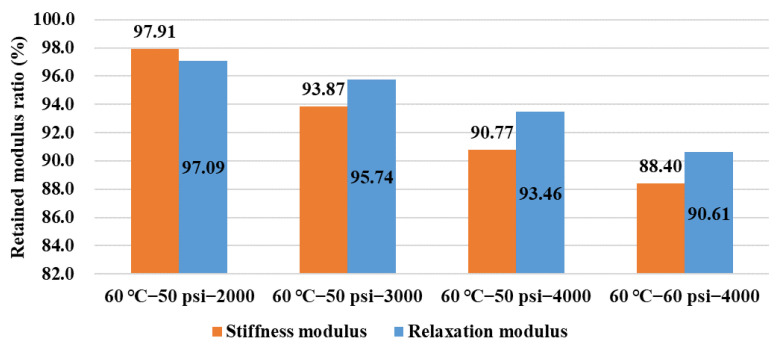
Variation of stiffness modulus and relaxation modulus in BBR tests.

The flexural creep behavior of asphalt binder before and after being conditioned in a dynamic pore water pressure environment was inspected using the BBR method at a low temperature of −12 °C. The flexural creep stiffness modulus *S* and relaxation modulus *E* are not the same, and the value of stiffness modulus *S* is usually obviously larger than relaxation modulus *E*, while their variation curves with time thus do not coincide completely in a BBR test [20]. The variations in retained stiffness modulus *S* and relaxation modulus *E* are shown in Figure 11. It can be clearly found that the retained modulus ratios for these two indicators declined with the parameters of conditioning duration and pore water pressure magnitude. Compared between the conditioning duration groups of 2000 and 3000 cycles in the dynamic water environment of 60 °C−50 psi, the percent change in the stiffness modulus and relaxation modulus were 2.09% and 2.91% in the group of 2000 cycles, respectively, but this value relationship was reversed in the group of 3000 cycles, which were 6.13% and 4.26%, respectively. In this case, more rheological behaviors can be explored for asphalt binder based on different indicators, such as relaxation modulus in this study.

**Figure 12:**
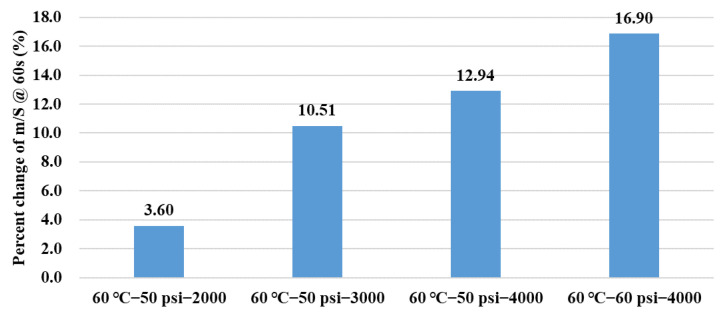
Percent change of *m*/*S* at 60th s in BBR tests.

Compared with the virgin asphalt binder, the indicator *m/S @ 60th* increased with conditioning duration and pore water pressure magnitude, and their percent change ratios also exhibited an increasing trend, as shown in Figure 12, which indicates a more severe erosion effect in a harsh dynamic pore water pressure environment. It can be concluded that the indicator *m/S @ 60th* can well evaluate the erosion degree of asphalt binder before and after being conditioned in a dynamic water environment.

**Figure 13:**
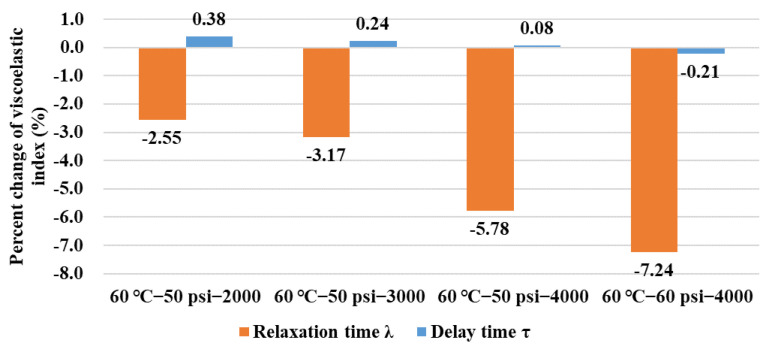
Percent change of viscoelastic indexes in BBR tests.

The percent change in the viscoelastic indicators is shown in Figure 13. It can be found that the relaxation time *λ* of the asphalt binder continually shortened after being conditioned in the dynamic pore water pressure environment, and its increasing variation trend is obvious with the increase in conditioning duration and environment severity. Delay time *τ* slightly increased after being conditioned, but its variation trend in percent change ratio showed a reversal phenomenon in the 60 °C−60 psi group. It indicated a slightly more viscous performance for the asphalt binder after being conditioned in the dynamic pore water pressure environment. The resistance ability to moisture damage of an asphalt mixture, such as HMA, highly counts on the combined performance of its components, which include asphalt binder and fine aggregate mixture (FAM). After being eroded by a dynamic water environment, although the asphalt binder component exhibited a slightly improved viscous performance, the FAM component was eroded greatly, which together induced the deterioration in comprehensive performance for HMA [12]. To further explore the obvious change in viscoelastic behavior for asphalt binder, it is necessary to set a larger interval for the parameter of conditioning duration in a future study.

### 4.4. Damage Mechanism Analysis via FTIR Spectrums

**Figure 14:**
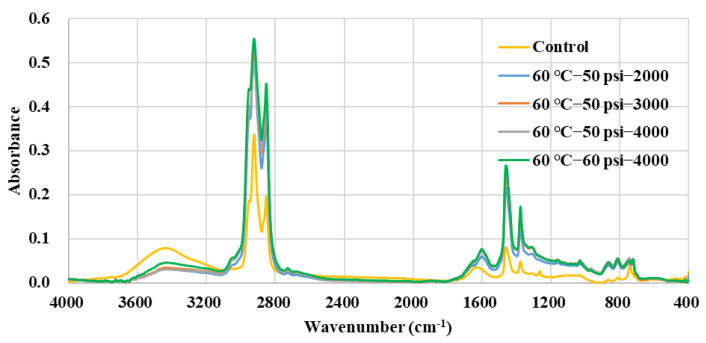
FTIR spectra of different types of asphalt binder.

The FTIR absorbance spectra of the five types of asphalt binder samples which were conditioned in the different water environments of dynamic pore water pressure are summarized in Figure 14. Obvious characteristic peaks can be found in several of the same ranges of wavenumbers, such as 3600–3200, 3100–2800, 1800–1200, and 1000–600 cm^−1^, and the absorbance curves of the asphalt binder exhibited basically regular fluctuations with the increase in conditioning duration and environmental severity [21]. For example, the control group showed a relative higher absorbance value in the wavenumber range of 3600–3200 cm^−1^ but held lower absorbance values in other wavenumber ranges. The group of 60 °C−60 psi−4000 cycles basically showed the highest absorbance values within the wavenumber range of 3000–1200 cm^−1^.

**Figure 15:**
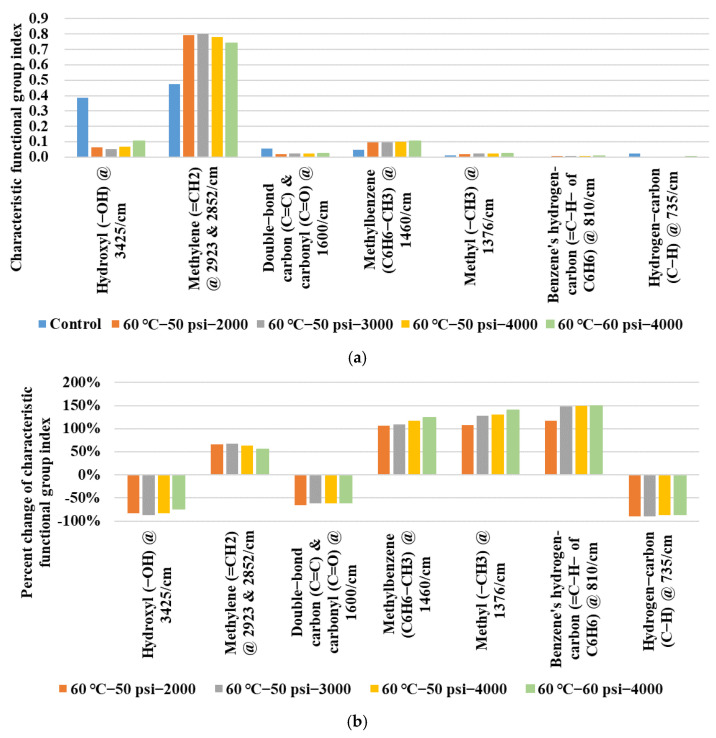
Characteristic functional group index of different types of asphalt binder: (**a**) Characteristic functional group index; (**b**) Percent change in characteristic functional group index.

The functional group index *I_i_* was applied to quantitatively evaluate the comprehensive features of the different obvious characteristic peaks, which could help to explore the change in the detailed functional groups related to the moisture damage of asphalt binder. Obvious characteristic peaks with their functional group indexes are summarized in Figure 15a. It could be clearly found that the methylene group (=CH_2_) held the largest absolute values of the functional group index which were mainly due to the consideration of the two characteristic peaks of 2923 and 2852 cm^−1^. For the hydroxyl group (−OH) at 3425 cm^−1^, great declines in the absolute values of the functional group index occurred inside the asphalt binder after water conditioning, while unremarkable absolute values of *I_i_* for the other functional groups appeared as shown in Figure 15a. 

Compared with the control group, the percent changes in the functional group indexes of the water-conditioned asphalt binder were further calculated to quantify the detailed changes. As shown in Figure 15b, the increase (percent change in *I_i_* > 0) and the decrease (percent change in *I_i_* < 0) in the functional group index values for the different functional groups can be clearly seen. Great changes occurred in methylbenzene (C_6_H_6_−CH_3_) @ 1460 cm^−1^, methyl (−CH_3_) @ 1376 cm^−1^, and benzene’s hydrogen-carbon (=C−H− of C_6_H_6_) @ 810 cm^−1^, because their percent changes in *I_i_* were larger than 100%, and their growth degree basically increased with conditioning duration and environmental severity. Similarly, the decrease in *I_i_* indexes for the hydrogen-carbon bond (C−H) @ 735 cm^−1^, double-bond carbon bond (C=C) and carbonyl groups (C=O) @ 1600 cm^−1^ also exhibited this kind of trend. For the percent changes in the *I_i_* indexes in the hydroxyl group (−OH) @ 3425 cm^−1^ and methylene group (=CH_2_) @ 2923 & 2852 cm^−1^, their fluctuations showed a different trend which increased first but decreased later with conditioning duration and environmental severity. It might be one of the possible reasons why dynamic water environment conditioning made a trend of non-monotonic change on the asphalt binder’s rheological behaviors.

## 5. Conclusions

This study assessed the rheological behaviors and the damage mechanism of asphalt binder under the erosion of a dynamic pore water pressure environment using the DSR, BBR, and FTIR methods. Based on the analysis discussed above, the following conclusions can be obtained:

(a) A longer conditioning duration is beneficial for the asphalt binder to recover its adhesion at the high temperature of 60 °C in MSCR tests, but the increasing pore water pressure magnitude of 60 psi held an opposite effect in this study.
(b) The fatigue life of the asphalt binder at the medium temperature of 20 °C in the LAS tests decreased obviously with the conditioning duration and environmental severity of the dynamic pore water pressure, but the reducing rate gradually slowed down, while the groups of 50 psi−4000 cycles and 60 psi−4000 cycles held a comparable erosion effect.
(c) The flexural stiffness modulus and relaxation modulus at the low temperature of −12 °C in the BBR tests exhibited an obvious decreasing trend with conditioning duration and environmental severity. The erosion effect on the asphalt binder was gradually enhanced, but it also exhibited a slightly more viscous performance.
(d) Dynamic water environment conditioning induced obvious appearances of several main characteristic peaks in the FTIR absorbance spectra of the asphalt binder. The functional group indexes presented a trend of non-monotonic change with conditioning duration and environmental severity, which made the asphalt binder show complicated rheological behaviors such as non-monotonic variations in performance and the abnormal improving effect induced by dynamic pore water pressure conditioning.

## Figures and Tables

**Table 1:** Physical properties of the base asphalt binder sample.

Items		Units	Requirements	Results
Penetration (25 °C, 100 g, 5 s)		0.1 mm	80–100	85
Softening point		°C	≥45	48.5
Ductility (5 cm/min)		cm	≥45 (10 °C)	53
Residue after rolling thin film oven test (163 °C, 85 min)	Mass loss	%	≤±0.8	−0.450
	Residual penetration ratio (25 °C, 100 g, 5 s)	%	≥57	65
	Residual ductility (5 cm/min)	cm	≥8 (10 °C)	20
Performance Grade (PG)		-	64–16	

## Data Availability

Not applicable.
